# Incidence and causes of cancellations of elective operation on the intended day of surgery at a tertiary referral academic medical center in Ethiopia

**DOI:** 10.1186/s13037-018-0171-3

**Published:** 2018-08-27

**Authors:** Melaku Desta, Addissu Manaye, Abiot Tefera, Atalay Worku, Alemitu Wale, Alemlanchi Mebrat, Negesso Gobena

**Affiliations:** 1 College of Health Science, Department of midwifery, Debre Markos University, PO. Box: 269, Debre Markos, Ethiopia; 2 College of medicine and Health Science, Department of Anesthesia, Hawassa University, Hawassa, Ethiopia; 3 College of Medicine and Health Sciences, Lecturer and Senior Anesthetist, Hawassa University, Hawassa, Ethiopia

**Keywords:** Cancellation, Elective surgery, Operating theatre, Ethiopia

## Abstract

**Background:**

Elective surgical case cancellation refers to any elective surgical case that is the list on the day prior to surgery but not operated upon as scheduled. Case cancellation has a major cause of psychological trauma to patients and their families. Despite little is known in Ethiopia. Therefore, this study aimed to assess incidence and reasons of cancellations of elective operation on the intended day of surgery at tertiary referral academic medical center in Ethiopia.

**Methods:**

A prospective hospital-based cross-sectional study design was conducted in a tertiary referral academic medical center in Ethiopia among 146 participants. A self-administered questionnaire with an observatory checklist was used for collecting data from the anesthetist, nurse, and surgeons.

**Result:**

In this study, 462 patients were scheduled for elective surgical operations. Among those, nearly almost one-third 146 (31.6%) of the operations were cancelled and 316 (68.4%) patients were operated on their planned date. The most common reason for cancellation were surgeon related (35.8%), patient related (28.7%), management related (21.2%) and anesthesia related factors (14. 4%). The cancellation was mainly due to improper scheduling (20.5%%), unavailability of surgeons (8.9%), unavailability of oxygen and blood (8%) and equipment (5.5%). Orthopedic (28.8%) and general surgery (17.1%) were the commonest cancelled cases.

**Conclusion:**

The cancellation rate in our academic medical center remains high. Improper scheduling, unavailability of surgeons, medical illness, and unavailability of operating room equipment were the commonest reason for the cancellation of elective operation. Most cancellations were preventable. For this, proper preoperative assessment, proper scheduling, fulfilling necessary operating room equipment’s and cross-matched blood by the hospital and other stakeholders, early clear communication with operating room team like surgeons was recommended.

## Back ground

Cancellation of elective operation on the intended day of surgery (DOS) is considered when a patient’s name has appeared on the list for surgical operations but the operation was not done on the intended scheduled date [1]. DOS cancellations are a world-wide problem, ranging from 0.37–28% in developed [2–9] and from 11 to 44% in developing countries [10–14]. It can be avoidable and non-avoidable [15] and avoidable cancellations were the commonest [4, 16, 17].

Cancellation of elective operation decrease the over-all efficiency of the operating rooms(ORs), reduces utilization of OR time and waste resources [18], subsequently endup with high economic burden for the patients and hospitals associated with extended hospital stay and repeated pre-operative preparations [12, 16, 19–21]. Hence, OR generates the highest costs and the largest source of revenues for the hospitals [22]. It affects surgeon productivity and staff morale and also causes psychological trauma or distress for the patients and as well the families [20, 23].

There are different reasons of cancellation of elective surgery that varies from one hospital to another [24]. The range of reasons given include inadequate pre-op assessment and preparation [24–27], management related factors [13] or infrastructural limitations [12, 17], lack of operating room time [6, 28], and unavailability of hospital beds [3], patient-related factors [2, 4, 15], surgery related factors (surgeon related issues, improper scheduling [29] and Anesthesia related factors [30].

The most common reason of those cancellations was preventable [16].Despite most cancellations are avoidable and many improvements done for patient quality of care, cancellation of elective surgery is high in Ethiopia [29]. For this, an efficiency of the operating room theatre needs to be permanently and universally improved through addressing of information and reduction of cancellation of elective surgical procedures. However; the study is scarce in Ethiopia in the study area of interest. Therefore, this study was aimed to assess incidence and causes of cancellation of elective operation on the intended day of surgery at a tertiary referral academic medical center. The finding of this study will assist us in making to enhance efficiency and minimize wastage of already limited hospital resources and manpower, policymakers or planners to set their target with feasible interventions in the study area, as a baseline for further studies and might be a means of achieving the Sustainable Development Goals (SDGs).

## Methods

### Study design and setting

A prospective Hospital-based cross-sectional study was conducted in Hawassa university comprehensive specialized hospital, which is a tertiary referral academic medical center found in Hawassa town, one of the regional city in Ethiopia (it is the capital city of SNNPR) located at about 276 km from Addis Ababa. The hospital was built around 1996E.C and it serves as a regional referral hospital for a long period of time and now it becomes a comprehensive specialized hospital one year back.

The hospital has four major wards with their perspective OPD and also minor wards with over 400 beds and provides the high quality of patient care in a broad range of services to over 90, 200 outpatients, 18,116 hospitalized patients and 1092 emergency cases annually. The hospital has one main operation theatre, which has six rooms. These rooms are used for general surgery, orthopedic surgery, gynecologic surgery, urologic& neurology and plastic surgery for both inpatient and outpatient. It was conducted from March 1–20, 2018.

### Study participants and sampling procedure

All patients who admitted to the tertiary referral medical center for elective surgical procedures from March 1–30, 2018 were the source population. All patients who were scheduled to undergo routine elective surgical operation, but due to some reasons they could not have their surgery done were included and patients scheduled in a minor operating room for minor surgery, which does not have preoperative assessment sheet and emergency cases were excluded. The interview was started by selecting a random sample.

### Variables and measurement

The dependent variable of the study was the cancellation of operation and the independent variables included were age, sex and ASA class, the type of operation canceled and reasons for cancellation. Cancellation of operation was defined as any elective operation that was either scheduled on the final theatre list for that day or was subsequently added to the list, and that was not performed on that day. The reasons for cancellation were categorized as hospital-related, surgeon-related, and patient-related and anesthetist related factors.

### Data collection technique, quality control

A structured questionnaire was adapted from a validated and modified individual patient questionnaire and other related literature. Data on the canceled operations were obtained from the daily operating theatre list and documented in a special form. A pre-tested was done on 5% of the study subjects on other nearby health facility and training for 3 days was given. The causes for cancellation were provided by either the surgeon or the resident and prospectively recorded into the computerized database. The assigned OR staff confirmed the cancellation reason and added additional explanation if necessary by calling patients or through direct inquiry of clerical and clinical staff the following day. A questionnaire was prepared in English and translated into the local language and finally to English. Five Anesthesia students were collecting the data and one senior staff was recruited as supervisors. The data were checked on the daily basis for completeness and consistency.

### Data processing and analysis

The data were checked, cleared, entered and analysised by using SPSS version (20.0). Descriptive and basic analytical statistics were used to summarize the data. Data were presented in the form of proportions, frequency tables and using bar graph. Means and standard deviation The analysis was done by reporting of STROBE statement checklist [31].

### Ethical considerations

Ethical clearance and permission were obtained from Hawassa University, College of Medicine and Health Science Institutional review board and permission was secured from medical center director. Informed consent was obtained and confidentiality was ensured.

## Results

### Sociodemographic demographic characteristics

A total 462 elective surgical cases were scheduled for operation during the study period. The mean age of the participant was 26.7 ± 4.37(SD) years and a majority of the participants were in the age group 21–30 years. From the study 316(68.4%) were operated on the intended day of surgery and 146(31.6%) were cancelled. From the cancelled cases 83(56.8%) were male and 61(43.2%) were female. Male to female ratio was 1.31:1. Male have a high rate of cancellation than female (Table 1).

**Table 1:** Sociodemographic characteristics of cancelled cases (*n* = 146) at a tertiary referral academic medical center in Ethiopia, 2018

Variable	Category	Frequency	Percent
Age	less than 10	25	17.1
	11–20	21	14.4
	21–30	34	23.3
	31–40	29	19.9
	41–50	17	11.6
	51–60	12	8.2
	61–70	6	4.1
	greater than 71	2	1.4
Sex	Male	83	56.8
	Female	63	43.2
ASA Class	ASA C-I	87	59.6
	ASAC-II	53	36.3
	ASA C III and above	6	4.1

*ASA* American Society of Anesthesia

### Causes of cancellation of elective operation

**Fig. 1:**
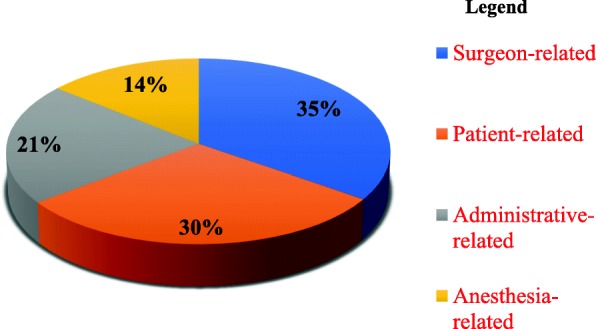
Specific causes of cancellation of elective surgery in at a tertiary referral academic medical center in Ethiopia, March 1–20, 2018

The most frequent causes for elective case cancellation were surgeon-related factors, which accounts for 51 cases (35.8%) followed by patiently-related reasons, (30%) [Fig. 1]. Regarding, the surgeon related reasons, scheduling an emergency procedure (11.6%) followed by over scheduling of elective cases (10.9%) and unavailability of the surgeon (10.3%) were the commonest reason of cancellation of elective surgery.

The highest frequent patient-related reasons for elective case cancellation were patients who have acute and chronic medical illness (11.6%) and the leading reason is patient on medication for the treatment of this illness. Hypertension (35.3%), diabetes mellitus (23.5%), Ischemic heart diseases (17.6%), urinary tract infections and other illnesses each account, (11.8%) were the commonest illness. Administrative-related cause of cancellation of elective cases is primarily by lack of oxygen and blood (8%) (Table 2**).**

**Table 2:** Reasons of cancellation of elective surgery at a tertiary referral academic medical center in Ethiopia, 2018

Reason of cancellation	Category	Frequency (%)
Patient-related	Refusal to surgery	6(4.1)
	patient on medication	10(6.8)
	Acute and chronic medical illness	17(11.6)
	patient not fasting	7(4.8)
	patient not paid	4(2)
	failure to arrive	6(4.1)
Surgeon-related	surgeon unavailability	15(10.3)
	diagnosis change	5(3.4)
	patient require other surgical workup	9(6.2)
	emergency scheduling	17(11.6)
	over scheduling of elective surgery	16(10.9)
Administrative- related	no availability of or equipment	8(5.5)
	delayed laboratory test	6(4.1)
	Lack of oxygen and blood	12(8.0)
	Unavailable of bed	8(5.5)
Anesthesia -related	patient unfit for anesthesia	10(6.8)
	Abnormal lab result	5(3.4)
	Unavailable equipment	3(2.1)
	Difficult intubation	4(2.7)

### Cases cancelled among elective operations in each department

**Fig. 2:**
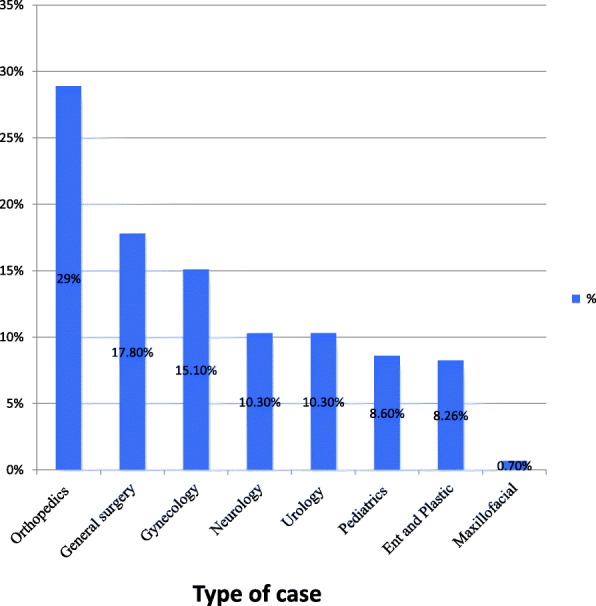
Cases cancelled among elective surgery in each department at a tertiary referral academic medical center in Ethiopia, March 1–20, 2018

Orthopedic was the department with the high rate of elective surgical cases scheduled 109 cases (23.6%)from the total schedule and has high cancellation rate from the other department 42 cases (28.8%) followed by general surgery which accounts 26 cases (17.1%). The least cancellation rate was observed from maxillofacial department1 cases (0.6%) [Fig. 2].

## Discussion

Cancellation of elective surgical operations in hospitals is a significant problem with many undesirable consequences. Cancellations are a major drain on health resources, increases theatre costs, decrease patient satisfaction, wasted operating room time and decrease efficiency. An efficient surgical service should have a low rate of cancellation [12, 16, 20].

The study revealed that about 31.6% of the elective surgical case was canceled, indicates preoperative system was inefficient. The result of the study is in line with studies done in India, 30.3% [32] and Burkina Faso, 36.7% [13] and Nigeria, 28% [1]. But, this finding is higher than when compared to other studies done in America, 4.4% [4], Brazil 6.8% [9], German, 12.7% [33], Wales,7.6 [6], Tanzania, 21% [12], New delhi,17.6% [28], Sudan, 20.2% [11], and Jimma University, 23% [29]. This discrepancy might be due to the fact that in sociodemographic characteristics, sample size, study area, and methodological difference. Beyond this the operation theatre of this hospital being on innovation, lack of operating room equipment’s and bed, unavailability of surgeon, and is the largest university specialized hospital at the regional level, results in higher case flow and referral cases, results decreasing operating room time and giving priority for emergency referral cases subsequently end up increased the number of cancelled cases.

21–30 years old age group were the highest canceled age groups (23.3%) followed by 31–40 years old group (19.9%) canceled groups and less frequent cancellation were over the age of seventy (1.4%) and in Spain cancellations [34] were more common in patients aged 0–10 years (13%) followed by those aged 21–30 years (9%). Cancellations were less frequent in older age groups (5% among 71–80 years and 6% among 61–70 years [16]. But in Sudan [11], the highest canceled group was 61–70 years old 31.1% followed by 51–60 years old group 25.4% [18]. The difference might be due to those age groups; aged 0–10 and older age groups might be more likely assessed preoperatively before the day of surgery and given priority for them.

Surgeon-related factors were the most common cause for cancellation of elective surgical cases in the hospital, which accounted for 51(35.8%) of cases. The commonest reason of surgeon related factor was scheduling of emergency surgery (11.6%), scheduling of elective surgery (10.9%) unavailability of a surgeon (10.3%) and change of diagnosis is the least cause. This is supported by a study done in Spain [34], Hong Kong [6] and India [32], Burkina Faso [13] and Jima university [29], surgeon-related factors due unavailable operating time and unavailability of surgeons or inappropriate scheduling were the leading reason of elective surgical case cancellation. This is might be due to the fact that unavailability of surgeon and scheduling of emergency surgery is known the case for postponed the elective case on the day of surgery. Over scheduling of elective surgery might be more likely result in lack of available operating room time, results in cancellation of an elective case on the day of surgery.

This study revealed that patient-related factors were another common reason for the cancellation of elective surgical cases accounted (30%) of canceled cases. This study is similar to other studies done in Brazil [9], India [32] and Burkina Faso [13], patient-related factors were the common cause for cancellation. Cancellation of the case caused by patient related factor was mainly due to acute and chronic medical illness and being on medication. This might have explained due to that those patients who haven’t stop medications prior to the day of surgery according to the illness might affect the surgical procedure and action of the anesthetic agent.

Furthermore, administration related reason accounts for 21.2% for cancellation, the commonest were failed to prepare cross-matched blood 12(8%) followed by OR material shortage and bed unavailability 8(5.5%). The finding is similar with compared to studies done at china [6], Saudi Arabia [35] and Sudan [11]. It is not surprisingly if the operating time is not fulfilling necessary equipment it delays operating room time and without cross-matched blood, difficult to do surgery. Hence, there might be bleeding, end up severe postoperative anemia and shock result cancellation of an elective surgical case.

Moreover, the study showed that orthopedic cases (28.7%) followed by general surgery (17.8%) and gynecology surgery (15.1%) was the commonest and maxillofacial (0.6%) was the least cancelled cases. This finding is supported by other similar studies conducted in Hong Kong [6], Saudi Arabia [35], orthopedics account, 33.9%) and in Finland [36], 31.8% orthopedics cases was the commonest canceled elective surgical procedure. This might be due to the fact that high rate of trauma and road traffic accident in the study area leads to giving priority for emergency orthopedic cases to maintain ongoing blood loss. Beyond this, it might have explained due to the fact that lack of cross-matched blood results in high rate of cancellation. Hence, orthopedic, gynecology, and general surgeries are the invasive procedure, which is more likely at risk of excessive bleeding.

This study can’t make causal relationship (difficult to know which precede the exposure or outcome hence it is cross-sectional descriptive study and less likely to generalize to the general population hence it is a health facility based. In addition to this, there might be a recall bias, and being a self reported study might not give the exact figure of the cause of cancellation.

## Conclusions

The rate of cancellation in this medical care center remains high. This study showed that the majority of cancellations were deemed avoidable and hospital related. Improper scheduling, unavailability of surgeons, acute and chronic medical illness and unavailability of operating room equipment were the commonest reason for the cancellation of an elective surgical case.

Cancellation of elective surgery can be prevented by simple steps and minimized if the patients with medical problems were detected early and referred for an anesthetic assessment soon after they are scheduled for surgery. Proper scheduling, provide adequate information for the scheduled patient, fulfilling necessary operating room equipment’s including cross-matched blood by hospital administrators and other stakeholders, clear communication with operating room team especially surgeons to be available on their schedule, and improve operating theater efficiency was recommended to reduce avoidable cancellation.

## Abbreviations

ASA: American Society of Anesthesiology
DOS: Day of Surgery
OPD: Outpatient Department
ORs: Operating Rooms
SDGs: Sustainable Development Goals
SNNPR: South nation nationality people representative
SPSS: Statistical package for the social science
